# Structural and Spectroscopic Effects of Li^+^ Substitution for Na^+^ in Li_x_Na_1-x_CaGd_0.5_Ho_0.05_Yb_0.45_(MoO_4_)_3_ Scheelite-Type Upconversion Phosphors

**DOI:** 10.3390/molecules26237357

**Published:** 2021-12-03

**Authors:** Chang-Sung Lim, Aleksandr Aleksandrovsky, Maxim Molokeev, Aleksandr Oreshonkov, Victor Atuchin

**Affiliations:** 1 Department of Aerospace Advanced Materials and Chemical Engineering, Hanseo University, Seosan 31962, Korea; 2 Laboratory of Coherent Optics, Kirensky Institute of Physics Federal Research Center KSC SB RAS, 660036 Krasnoyarsk, Russia; aleksandrovsky@kirensky.ru; 3 Institute of Nanotechnology, Spectroscopy and Quantum Chemistry, Siberian Federal University, 660041 Krasnoyarsk, Russia; 4 Laboratory of Crystal Physics, Kirensky Institute of Physics, Federal Research Center KSC SB RAS, 660036 Krasnoyarsk, Russia; msmolokeev@mail.ru; 5 Institute of Engineering Physics and Radioelectronics, Siberian Federal University, 660041 Krasnoyarsk, Russia; 6 Department of Physics, Far Eastern State Transport University, 680021 Khabarovsk, Russia; 7 Laboratory of Molecular Spectroscopy, Kirensky Institute of Physics, Federal Research Center KSC SB RAS, 660036 Krasnoyarsk, Russia; oreshonkov@iph.krasn.ru; 8 School of Engineering and Construction, Siberian Federal University, 660041 Krasnoyarsk, Russia; 9 Laboratory of Optical Materials and Structures, Institute of Semiconductor Physics, SB RAS, 630090 Novosibirsk, Russia; 10 Research and Development Department, Kemerovo State University, 650000 Kemerovo, Russia; 11 Department of Industrial Machinery Design, Novosibirsk State Technical University, 630073 Novosibirsk, Russia

**Keywords:** optical materials, chemical synthesis, molybdate, Raman spectroscopy, X-ray diffraction, phosphors

## Abstract

A set of new triple molybdates, Li_x_Na_1-x_CaGd_0.5_(MoO_4_)_3_:Ho^3+^_0.05_/Yb^3+^_0.45_, was successfully manufactured by the microwave-accompanied sol–gel-based process (MAS). Yellow molybdate phosphors Li_x_Na_1-x_CaGd_0.5_(MoO_4_)_3_:Ho^3+^_0.05_/Yb^3+^_0.45_ with variation of the Li_x_Na_1-x_ (x = 0, 0.05, 0.1, 0.2, 0.3) ratio under constant doping amounts of Ho^3+^ = 0.05 and Yb^3+^ = 0.45 were obtained, and the effect of Li^+^ on their spectroscopic features was investigated. The crystal structures of Li_x_Na_1-x_CaGd_0.5_(MoO_4_)_3_:Ho^3+^_0.05_/Yb^3+^_0.45_ (x = 0, 0.05, 0.1, 0.2, 0.3) at room temperature were determined in space group *I*4_1_/*a* by Rietveld analysis. Pure NaCaGd_0.5_Ho_0.05_Yb_0.45_(MoO_4_)_3_ has a scheelite-type structure with cell parameters *a* = 5.2077 (2) and *c* = 11.3657 (5) Å, *V* = 308.24 (3) Å^3^, *Z* = 4. In Li-doped samples, big cation sites are occupied by a mixture of (Li,Na,Gd,Ho,Yb) ions, and this provides a linear cell volume decrease with increasing Li doping level. The evaluated upconversion (UC) behavior and Raman spectroscopic results of the phosphors are discussed in detail. Under excitation at 980 nm, the phosphors provide yellow color emission based on the ^5^S_2_/^5^F_4_ → ^5^I_8_ green emission and the ^5^F_5_ → ^5^I_8_ red emission. The incorporated Li^+^ ions gave rise to local symmetry distortion (LSD) around the cations in the substituted crystalline structure by the Ho^3+^ and Yb^3+^ ions, and they further affected the UC transition probabilities in triple molybdates Li_x_Na_1-x_CaGd_0.5_(MoO_4_)_3_:Ho^3+^_0.05_/Yb^3+^_0.45_. The complex UC intensity dependence on the Li content is explained by the specificity of unit cell distortion in a disordered large ion system within the scheelite crystal structure. The Raman spectra of Li_x_Na_1-x_CaGd_0.5_(MoO_4_)_3_ doped with Ho^3+^ and Yb^3+^ ions were totally superimposed with the luminescence signal of Ho^3+^ ions in the range of Mo–O stretching vibrations, and increasing the Li^+^ content resulted in a change in the Ho^3+^ multiplet intensity. The individual chromaticity points (ICP) for the LiNaCaGd(MoO_4_)_3_:Ho^3+^,Yb^3+^ phosphors correspond to the equal-energy point in the standard CIE (Commission Internationale de L’Eclairage) coordinates.

## 1. Introduction

Complex molybdate crystals have become a subject of extensive investigation due to their diverse crystal chemistry, high chemical stability, and specific physical properties, which are promising for applications in such fields as electronics, laser systems, electrochemistry, and electro-photonics [1,2,3,4,5,6,7,8,9]. These lanthanide-activated double molybdates provide stable chemical and physical characteristics with excellent optical properties for favorable lanthanide admittance, relatively low phonon energy, and versatile applications in such fields as solar cells, lasers, biomedical and optoelectronic devices, optical sensors, etc. [10,11,12,13,14,15,16]. Many molybdate crystals are appropriate for the incorporation of rare earth (Ln) ions in their structure, and the materials are considered as potential hosts for the creation of phosphors to be used in photonic structures [2,3,4,7,17,18,19,20,21,22,23]. Among such crystals, scheelite-type (ST) molybdates are particularly interesting in terms of the search for new structures, including structure-modulation effects, and promising spectroscopic characteristics [2,21,24,25,26,27,28,29,30]. Scheelites of general composition ABO_4_ (A = A^2+^ cation, B = Mo, W) crystallize in space group *I*4_1_/*a*. Generally, an ST structure is very stable and highly tolerant to mixed cation accommodation at the A position, which opens up the possibility for the creation of solid solutions with wide-range doping by Ln ions. In accordance with this, new binary and ternary molybdates with the ST structure and cation disorder at the A position were synthesized and their basic properties were evaluated [21,23,24,25,26,27,28,29,30,31]. Commonly, in combination with Ln^3+^ ions, such lower valence ions as Li^+^, Na^+^, Ag^+^, Ca^2+^, Sr^2+^, and Pb^2+^ are used to reach the average charge balance at the A position because these ions have appropriate effective radii [32].

In ST solid solutions, different combinations of Ln^3+^ ions can be reached without a loss of structural quality; this opens the way for the investigation of energy transfer (ET) effects in phosphor systems with binary and ternary doping [33,34,35,36,37,38,39]. In particular, the incorporation of appropriate Ln^3+^ pairs provides enhanced frequency upconversion (UC) properties under near-infrared laser excitation [21,29,30,31,33,34,35,36,38,39]. For Ho^3+^/Yb^3+^-doped UC phosphors, yellow emissions can be derived via a co-doping system based on the red emission bands from ^5^F_5_ → ^5^I_8_ transitions and green emission bands from ^5^S_2_/ ^5^F_4_ → ^5^I_8_ transitions [40,41,42,43]. Extremely wide color tunability was reported for Ho^3+^/Yb^3+^ UC phosphor upon the addition of Er^3+^ ions [44]. Laser active Ho^3+^ ions play the role of activator, and Yb^3+^ ions, as an efficient sensitizer with a high absorption cross section at 980 nm, could enhance the UC efficiency through the ET process between the activator and sensitizer induced by a unique energy level configuration.

The preparation of complex molybdates can be carried out via several specific processes [45,46,47,48,49,50,51,52,53,54,55]. Among the different methods, microwave accompanied sol–gel-based synthesis (MAS) can provide high-quality crystalline materials in a short processing period. The powder products fabricated by the MAS route are commonly characterized by a homogeneous microstructure and high chemical reproducibility, in reference to the nominal starting reagent ratio that is governed by the sol preparation step with high atom intermixing [56,57]. Compared with the usual methods, microwave synthesis has the advantages of shortening the reaction time and resulting in products with a small particle size, narrow particle size distribution, and high purity [58,59,60,61,62,63]. At the final step, the high-temperature microwave treatment efficiently stimulates crystal lattice formation [64,65].

Previously, a new division of triple ST molybdates NaTLn(MoO_4_)_3_ (T = Ca, Pb; Ln = La, Gd) was designed and prepared by the MAS process, and their properties, as promising phosphor hosts, were reported [21,31,64,65,66]. Also, the Li_x_Na_1-x_CaLa_0.5_Ho_0.05_Yb_0.45_(MoO_4_)_3_ solid solution was evaluated to determine the effect of Li^+^ substitution for Na^+^ on the frequency upconversion, and the ST structure was observed in the range of x = 0.05−0.4 [67]. To extend the capabilities of ST crystal engineering, it is interesting to reveal ions and ion combinations appropriate for incorporation at the mixed A position in complex ST crystals. In relation to this, the triple ST solid solutions (Li,Na)MLn(MoO_4_)_3_ (M = Ca, Sr, Pb) are of particular interest because the presence of Li^+^ ions can affect the photoluminescence properties of Ln^3+^ activators [67]. Thus, the present contribution is aimed at the preparation of Li_x_Na_1-x_CaLa(MoO_4_)_3_;Yb^3+^,Ho^3+^ compounds by the MAS-based method and evaluation of their spectroscopic properties. The influence of incorporated Li^+^ ions was investigated—to enhance the UC transition probabilities by the Ho^3+^ and Yb^3+^ ions in the ST molybdate Li_x_Na_1-x_CaGd_0.5_(MoO_4_)_3_—and is discussed in terms of the local symmetry distortion (LSD) around the cations in the substituted crystalline structure, The crystal structures and morphologies of the synthesized particles were evaluated by X-ray diffraction (XRD) and scanning electron microscopy (SEM), respectively. The spectroscopic characteristics were investigated under the consideration of efficient UC emissions, individual chromaticity points (ICP) according to Commission Internationale de L’Eclairage (CIE), and Raman scattering.

## 2. Experimental Section

In this experiment, molybdate solid solution Li_x_Na_1-x_CaGd_0.5_(MoO_4_)_3_: Ho^3+^_0.05_/Yb^3+^_0.45_ was designed to achieve efficient UC luminescent characteristics by variation of the Li/Na ratio (x= 0, 0.05, 0.1, 0.2, and 0.3) under fixed rare earth element contents of Gd^3+^ = 0.5, Ho^3+^ = 0.05, and Yb^3+^ = 0.45. Na_2_MoO_4_∙2H_2_O, Ca(NO_3_)_2_∙4H_2_O, Gd(NO_3_)_3_∙6H_2_O, (NH_4_)_6_Mo_7_O_24_∙4H_2_O, LiNO_3_, and Ho(NO_3_)_3_∙5H_2_O with 99.0% purity were purchased from Sigma-Aldrich, USA. Yb(NO_3_)_3_∙5H_2_O, with 99.9% purity, was purchased from Sigma-Aldrich, USA. Besides these, citric acid (CA) at 99.5% purity was obtained from Daejung Chemicals, Korea. Distilled water (DW), ethylene glycol (EG, A.R.), and NH_4_OH (A.R.) were used to bring about the transparent sol formation.

The sample notation introduced according to the nominal compositions is given in Table 1. Initially, for the sol preparation of (a) NCGM:HY, Ca(NO_3_)_2_∙4H_2_O at 0.4 mol%, Na_2_MoO_4_∙2H_2_O at 0.2 mol%, and (NH_4_)_6_Mo_7_O_24_∙4H_2_O at 0.171 mol% were dissolved in 80 mL 8M NH_4_OH with 20 mL EG. To make the sol of (b) LiNCGM:HY-0.05 for Li_0.05_Na_0.95_, Ca(NO_3_)_2_∙4H_2_O at 0.4 mol%, Na_2_MoO_4_∙2H_2_O at 0.19 mol%, LiNO_3_ at 0.01 mol %, and (NH_4_)_6_Mo_7_O_24_∙4H_2_O at 0.171 mol% were used. For the compositions of (c) LiNCGM:HY-0.1 for Li_0.1_Na_0.9_, (d) LiNCGM:HY-0.2 for Li_0.2_Na_0.8_, and (e) LiNCGM:HY-0.3 for Li_0.3_Na_0.7_, the reagent sets (c) Na_2_MoO_4_∙2H_2_O at 0.18 mol% and LiNO_3_ at 0.02 mol %, (d) Na_2_MoO_4_∙2H_2_O at 0.16 mol% and LiNO_3_ at 0.04 mol %, and (e) Na_2_MoO_4_∙2H_2_O at 0.14 mol% and LiNO_3_ at 0.06 mol % were applied. Subsequently, Gd(NO_3_)_3_∙6H_2_O at 0.2 mol%, Yb(NO_3_)_3_∙5H_2_O at 0.18 mol%, and Ho(NO_3_)_3_∙5H_2_O at 0.02 mol% were carefully weighed and dissolved very slowly in 100 mL of DW under slight heat treatment. Then, these two kinds of prepared solutions were slowly co-mixed and vigorously stirred. The CA/CM (molar ratio of CA to the total cation metal (CM) ions) was adjusted to 2:1. The intermixed solutions were 180–200 mL in volume, and they were heated slowly to ~80–100 °C in a 450 mL Pyrex glass. At this stage, the solutions were in a highly transparent state. Then, the solutions were subjected to the MAS-derived treatment. The typical procedure applied at this stage can be found elsewhere [21,31,64,65,66,67]. Then, the obtained dried black gels were ground and annealed at 800 °C for 16 h in air, with intervals of 100 °C between 600 and 800 °C. After the annealing process, pink-colored particles were obtained for the samples.

The structural properties of synthesized samples were evaluated by XRD analysis. The powder XRD patterns of the Li_x_Na_1-x_CaGd_0.5_(MoO_4_)_3_:Ho^3+^_0.05_/Yb^3+^_0.45_ particles for Rietveld analysis were precisely recorded over the angle range of 2θ = 5−90° at room temperature using a D/MAX 2200 (Rigaku in Japan) diffractometer with Cu Kα radiation and θ-2θ geometry. The 2θ size step was 0.02°, and the counting time was 5 s per step. The TOPAS 4.2 package was applied for the Rietveld analysis [68]. The typical microstructure and surface morphology of the obtained particles were observed using SEM (JSM-5600, JEOL in Japan) methods. The PL spectra were recorded at room temperature using a spectrophotometer (Perkin Elmer LS55 in UK). The Raman spectral measurements were performed using a LabRam Aramis (Horiba Jobin-Yvon in France) device with spectral resolution of 2 cm^−1^. The 514.5 nm line of an Ar ion laser was used as an excitation source and, to avoid sample decomposition, the power on the samples was kept at the 0.5 mW level.

## 3. Results and Discussion

**Figure 1:**
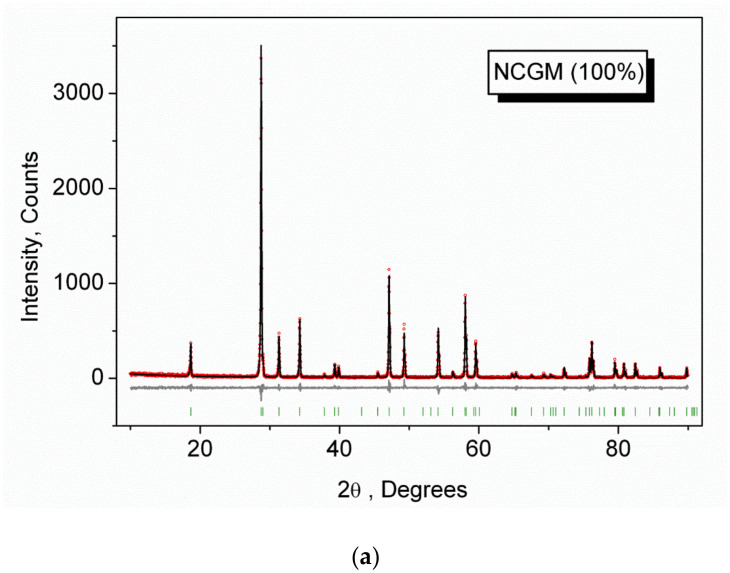

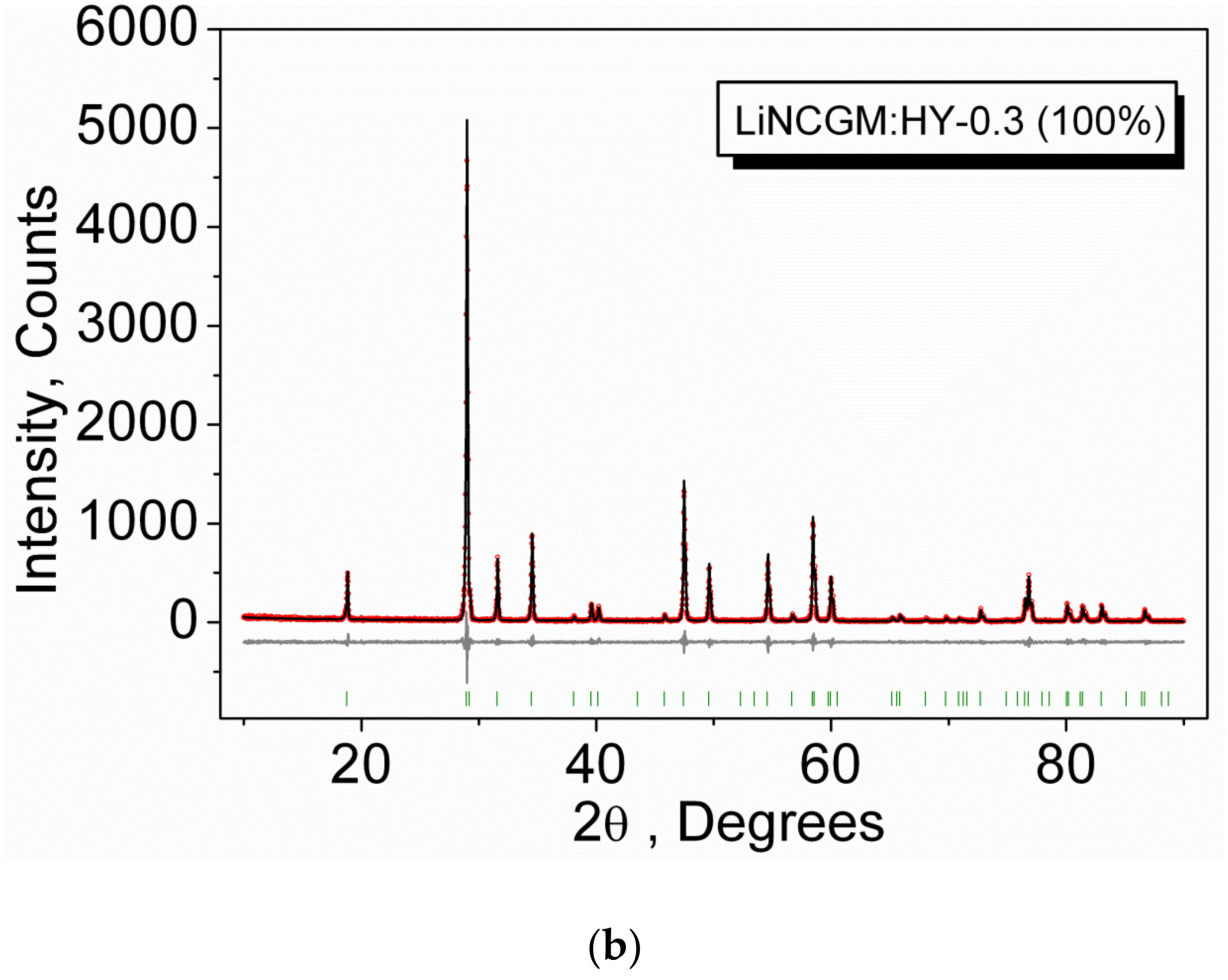
Difference Rietveld plots of (**a**) NCGM:HY and (**b**) LiNCGM:HY-0.3.

**Figure 2:**
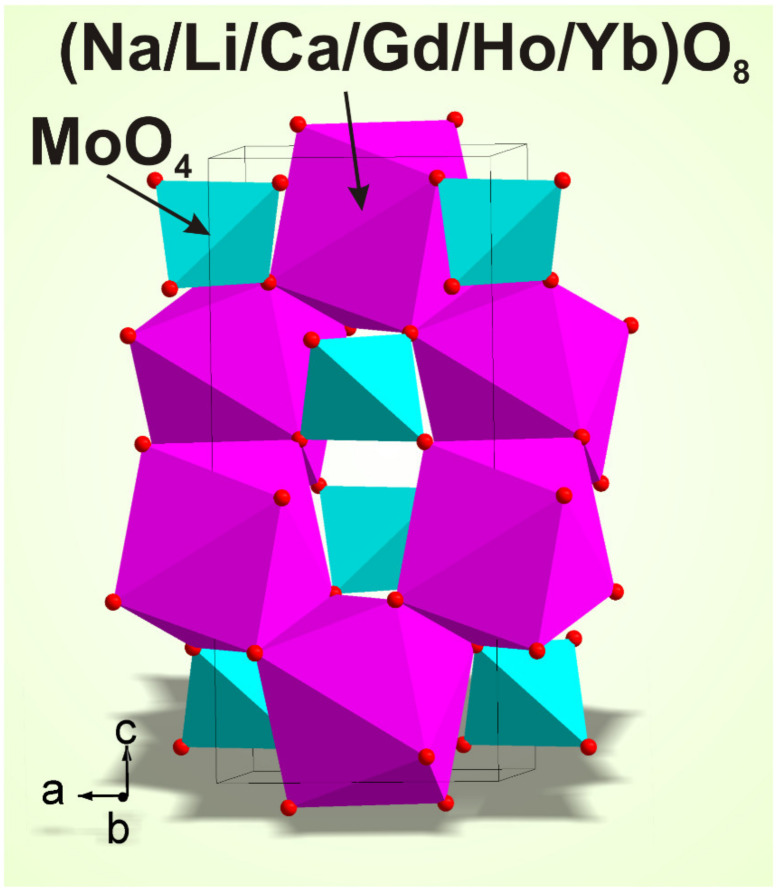
The crystal structure of LiNCGM:HY crystals. The unit cell is outlined. Lone atoms are omitted for clarity.

The XRD patterns measured for the samples listed in Table 1 are shown in Figure 1 and Figures S1–S3 (Supporting Information). In general, the patterns are similar. In Figure 1, for comparison, the XRD patterns of NCGM:HY (x = 0) and LiNCGM:HY-0.3 (x = 0.3) are presented. As can be seen, there is no significant difference even for the highest Li content. All peaks in all patterns were successfully indexed by the tetragonal cell (*I*4_1_/*a*) with cell parameters close to those of CaMoO_4_ [69]. Therefore, the crystal structure of CaMoO_4_ was taken as a starting model for Rietveld refinement. The Ca^2+^ ion site was considered as the one occupied by a mixture of Li^+^, Ca^2+^, Na^+^, Gd^3+^, Ho^3+^, and Yb^3+^ ions (Figure 2) with fixed partial occupations according to the nominal sample composition. The refinements were stable and gave low R-factors (Table 1, Figure 1 and Figures S1–S3). The atom coordinates and the main bond lengths are summarized in Tables S1 and S2, respectively.

**Figure 3:**
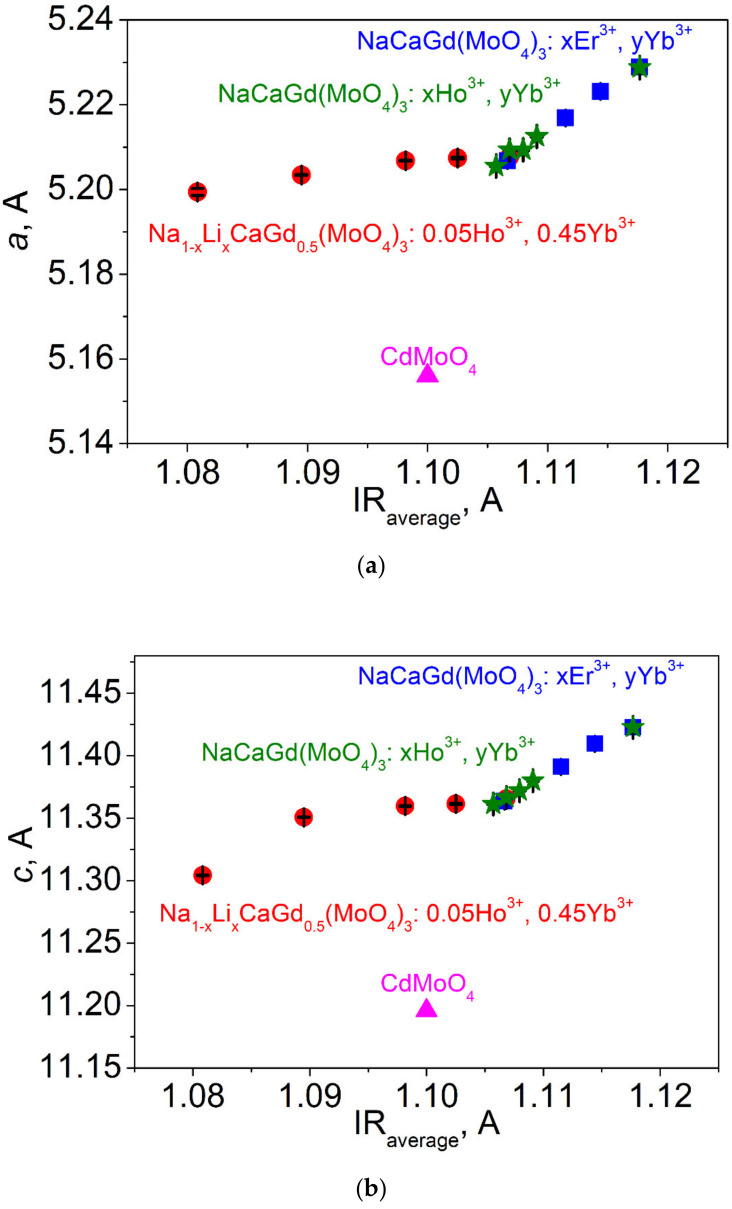

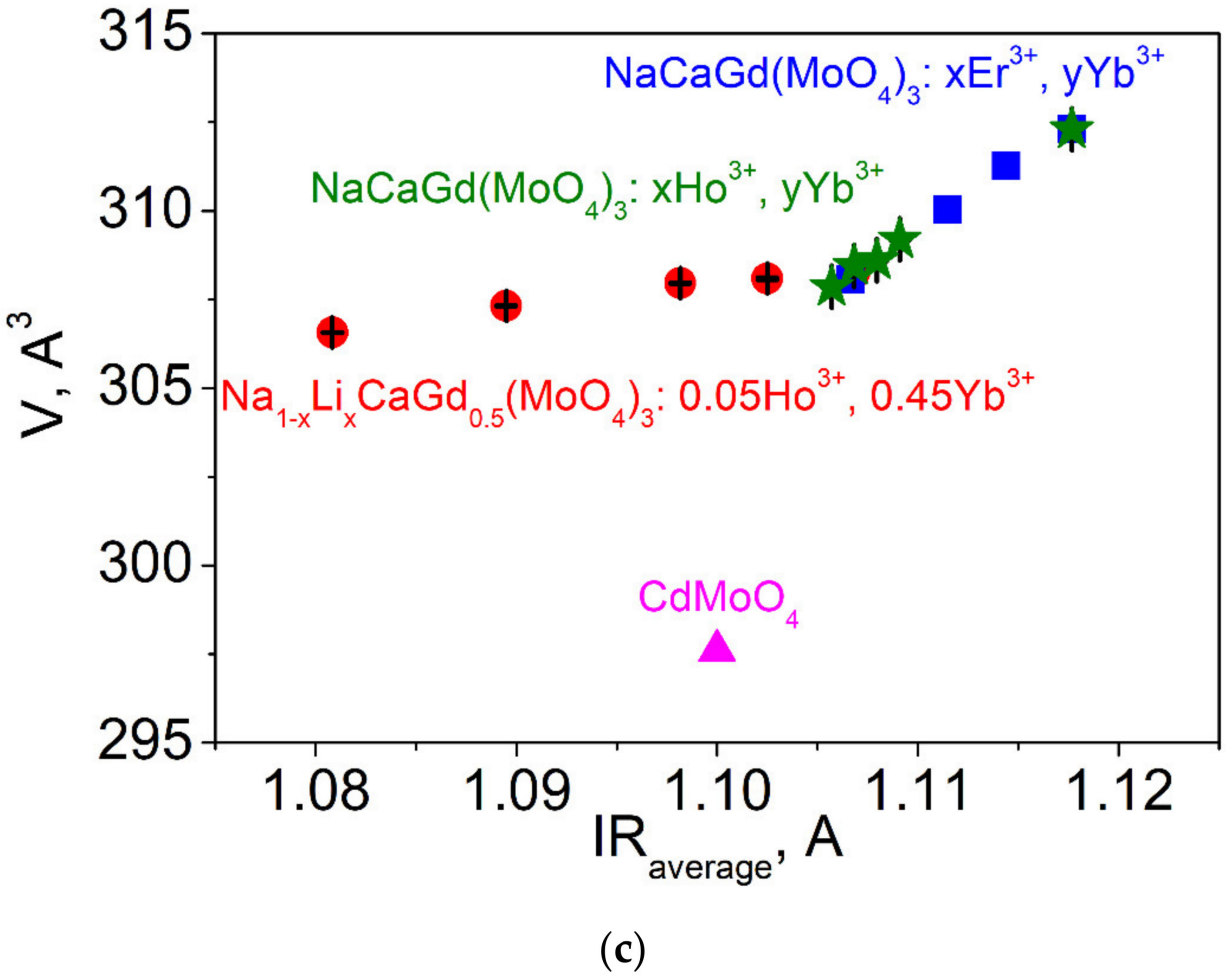
The dependence of (**a**) cell parameter *a*, (**b**) cell parameter *c*, and (**c**) cell volume *V* on the averaged ion radius IR(Li,Na/Ca/Gd/Ho/Er/Yb) in LiNCGM:HY and related scheelite-type molybdates.

**Figure 4:**
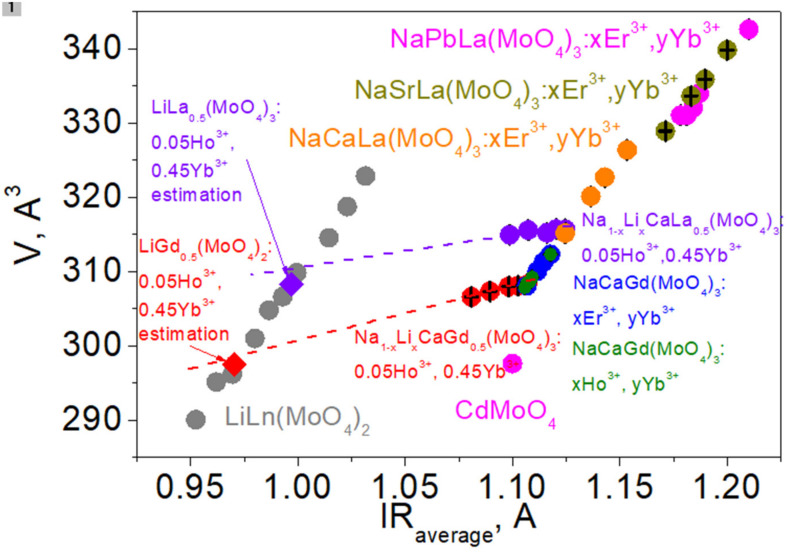
The dependence of unit cell volume *V* on the averaged ion radius IR(Li,Na/T/Ln) in Li_x_Na_1-x_TLn(MoO_4_)_3_ scheelite-type molybdates.

As is known, the effective radius of the Li^+^ ion is noticeably lower than that of the Na^+^ ion; therefore, substitution of Li^+^ for Na^+^ in the LiNCGM:HY compounds should induce a decrease in the average ion radius IR(Li/Na/Ca/Gd/Ho/Yb) of the A position and a related unit cell volume decrease in the ST structures. The dependencies of cell parameters and unit cell volume on IR(Li/Na/Ca/Gd/Ho/Yb) in the LiNCGM:HY compounds are shown in Figure 3. The IR values were calculated on the basis of the nominal compositions and the known system of ion radii [32]. For comparison, the same dependencies of two previously studied Gd-containing systems, NaCaGd(MoO_4_)_3_:Er,Yb and NaCaGd(MoO_4_)_3_:Ho,Yb [64,65], are also given in Figure 3. It should be pointed out that only the ST structure type was obtained in Refs. [50,51], and the structural parameters of NaCaGd(MoO_4_)_3_:Er,Yb and NaCaGd(MoO_4_)_3_:Ho,Yb remain unknown. For this reason, in the present work, the Rietveld refinement of the structures was implemented on the basis of the previously recorded XRD data [64,65]. The refinements were stable and gave low R-factors (Tables S3 and S4). From the observation of Figure 3, it is evident that, in the LiNCGM:HY compounds, the cell parameters and cell volume continuously decrease with decreasing IR(Li/Na/Ca/Gd/Ho/Yb) value or increasing Li content. This clearly proves the suggested chemical formulas of the LiNCGM:HY solid solutions. As to NaCaGd(MoO_4_)_3_:Er,Yb and NaCaGd(MoO_4_)_3_:Ho,Yb, with increasing doping level, the cell parameters and cell volume decrease proportionally to the IR value, according to the general trend observed in ST molybdates [66]. However, the decrease rate due to Li^+^ incorporation, instead of Na^+^, in LiNCGM:HY is strongly lower than that in NaCaGd(MoO_4_)_3_:Er,Yb and NaCaGd(MoO_4_)_3_:Ho,Yb due to the rare earth ion incorporation instead of Gd^3+^. Moreover, with increasing Li content, the points related to the LiNCGM:HY system move away from the point of CdMoO_4_, which is an ST molybdate with the lowest unit cell volume [70]. To verify this effect, it would be valuable to see the behavior in other related systems, and this is possible for Li_x_Na_1-x_CaLa_0.5_ (MoO_4_)_3_:Ho_0.05_Yb_0.45_ (LiNCLM:HY) [67]. To determine the structural parameters of the LiNCLM:HY solid solutions, Rietveld refinement was carried out for the LiNCLM:HY compounds on the basis of the previously reported XRD data [67]. The refinements were stable and gave low R-factors (Table S5). The obtained results are shown in Figure 4 in association with other known La-containing systems NaTLa(MoO_4_)_3_:Er,Yb (T = Ca, Sr, Pb) [21,31,66]. As can be seen, the variation in the unit cell volume with increasing Li^+^ content in the LiNCLM:HY crystals is nearly the same as that in LiNCGM:HY. Comparatively, NaTLa(MoO_4_)_3_:Er,Yb (T = Ca, Sr, Pb) solutions strongly follow the general trend of ST molybdates [66]. Thus, the substitution of Li^+^ for Na^+^ in LiNCGM:HY and LiNCLM:HY crystals generates a specific effect of unit cell compression that is not governed by the general trend of ST structures. It can be supposed that similar effects could be observed in other Li_x_Na_1-x_TLn(MoO_4_)_3_ solid solutions. Further details of the crystal structures of the compounds listed in Table 2 and Tables S3–S5 may be obtained from Fachinformationszentrum Karlsruhe, 76344 Eggenstein-Leopoldshafen, Germany (fax: (+49)7247-808-666; E-mail: crystdata@fiz-karlsruhe.de; http://www.fiz-karlsruhe.de/request_for_deposited_data.html) by quoting the deposition number: 2117686-2117704.

**Figure 5:**
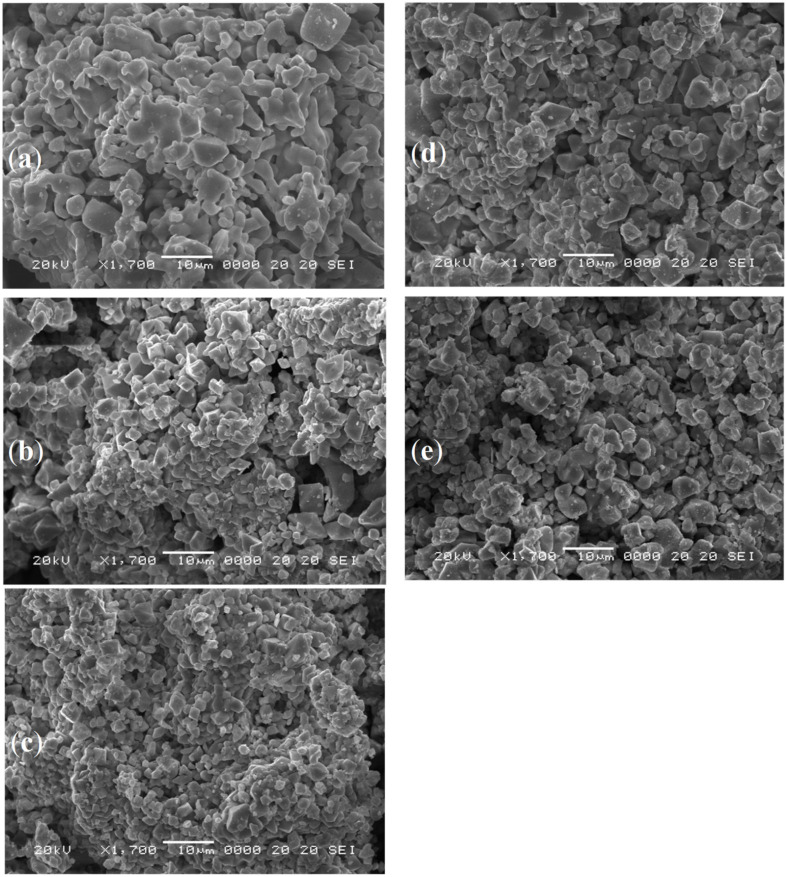
Scanning electron microscopy images of the synthesized (**a**) NCGM, (**b**) LiNCM:HY-0.05, (**c**) LiNCGM:HY-0.1, (**d**) LiNCGM:HY-0.2, and (**e**) LiNCGM:HY-0.3 particles.

The SEM images obtained for the representative compositions (**a**) NCGM:HY, (**b**) LiNCM:HY-0.05, (**c**) LiNCGM:HY-0.1, (**d**) LiNCGM:HY-0.2, and (**e**) LiNCGM:HY-0.3 are shown in Figure 5. As can be seen, the particle morphology is similar for all five samples. The samples contain uniform partly coalescent particles of 3–10 μm in size. However, the Li-containing samples presented in Figure 5b–e show a slightly smaller characteristic particle size of 3–5 μm, compared to that of the NCGM:HY sample (5–10 μm). Faceted forms were not detected, and this may be due to the comparatively short time of high-temperature treatment used in the MAS processing. In particular, agglomerated grains can be observed, and these could be induced by the material interdiffusion between the grains. Previously, similar grain agglomeration was observed in many other oxide materials subjected to high-temperature annealing [71,72,73]. Thus, MAS-derived synthesis, when applied to LiNCGM doped with Yb^3+^/Ho^3+^, provides powder products with an uniform micrograin morphology.

**Figure 6:**
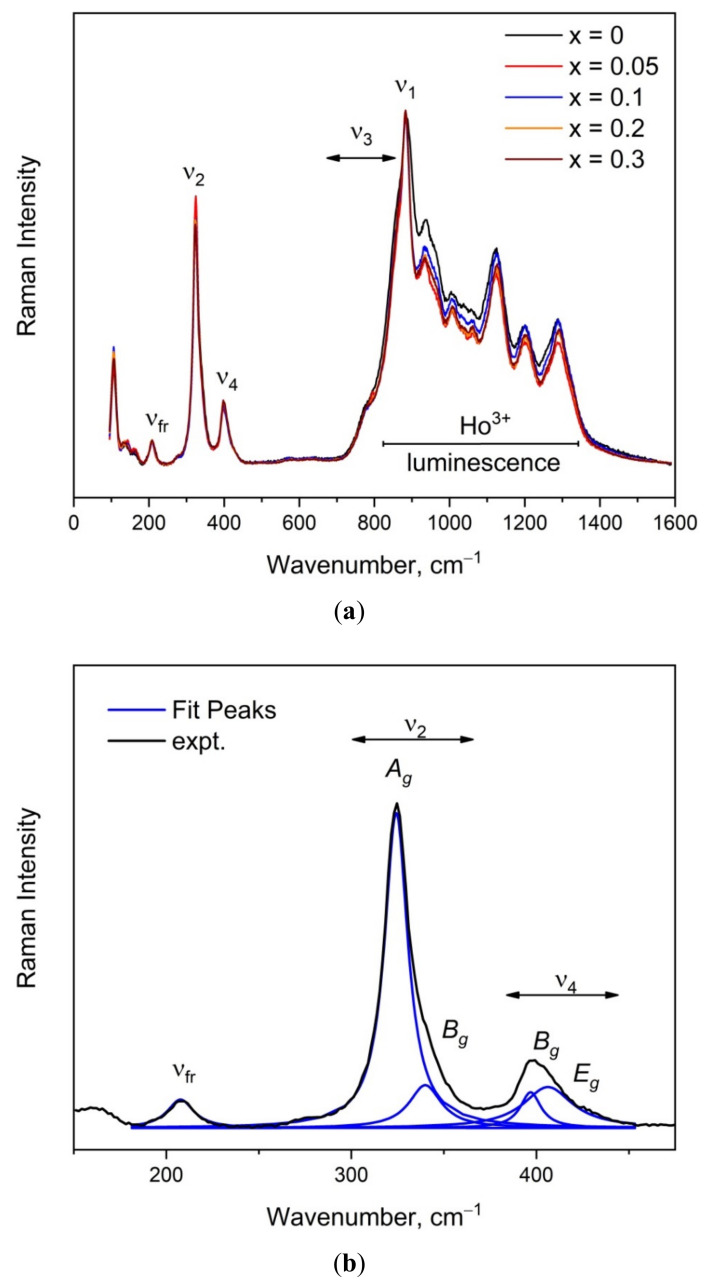
(**a**) Raman spectra of Li_x_Na_1-x_CaGd_0.5_(MoO_4_)_3_:Ho^3+^_0.05_/Yb^3+^_0.45_ (x = 0, 0.05, 0.1, 0.2, 0.3); (**b**) Raman spectrum decomposition in the range of MoO_4_ tetrahedra rotation and bending.

The Raman spectra recorded for Li_x_Na_1-x_CaGd_0.5_(MoO_4_)_3_:Ho^3+^0.05/Yb^3+^0.45 (x = 0, 0.05, 0.1, 0.2, 0.3) are shown in Figure 6a. As can be seen in Table S1, all of the investigated compounds contain only one crystallographically independent MoO_4_ tetrahedron, and these units occupy the sites with S_4_ point symmetry. It follows from the factor group analysis that Raman-active [MoO_4_]^2−^ ion vibrations can be listed as: *A_g_*, symmetric stretching; *B_g_* + *E_g_*, antisymmetric stretching; *A_g_* + *B_g_*, symmetric bending; and *B_g_* + *E_g_*, antisymmetric bending [28,74]. The strongest spectral band in Figure 6a at 884 cm^−1^ is related to the ν_1_ *A_g_* symmetric stretching vibration, and the ν_3_ antisymmetric stretching vibrations are located in the range of 720–860 cm^−1^. Part of the spectra between 820 and 1350 cm^−1^ is covered by the luminescence of Ho^3+^ ions, and the determination of exact positions of the peaks related to antisymmetric stretching vibrations is therefore impossible [3]. The Raman spectrum decomposition in the range of free MoO_4_ rotation and bending vibrations for Li_0.05_Na_0.95_CaGd_0.5_(MoO_4_)_3_:Ho^3+^0.05/Yb^3+^0.45 is presented in Figure 6b. The spectral contour in the range of 300–360 cm^−1^ consists of two lines related to symmetric stretching, and two lines were found in the range of 380–425 cm^−1^ (antisymmetric bending). The spectral peak at 208 cm^−1^ is shown with a single line and corresponds to the free rotation of MoO_4_ tetrahedra [75]. Thus, the number of observed peaks in this region is in accordance with the group theoretical analysis, confirming the high crystallinity of the samples.

**Figure 7:**
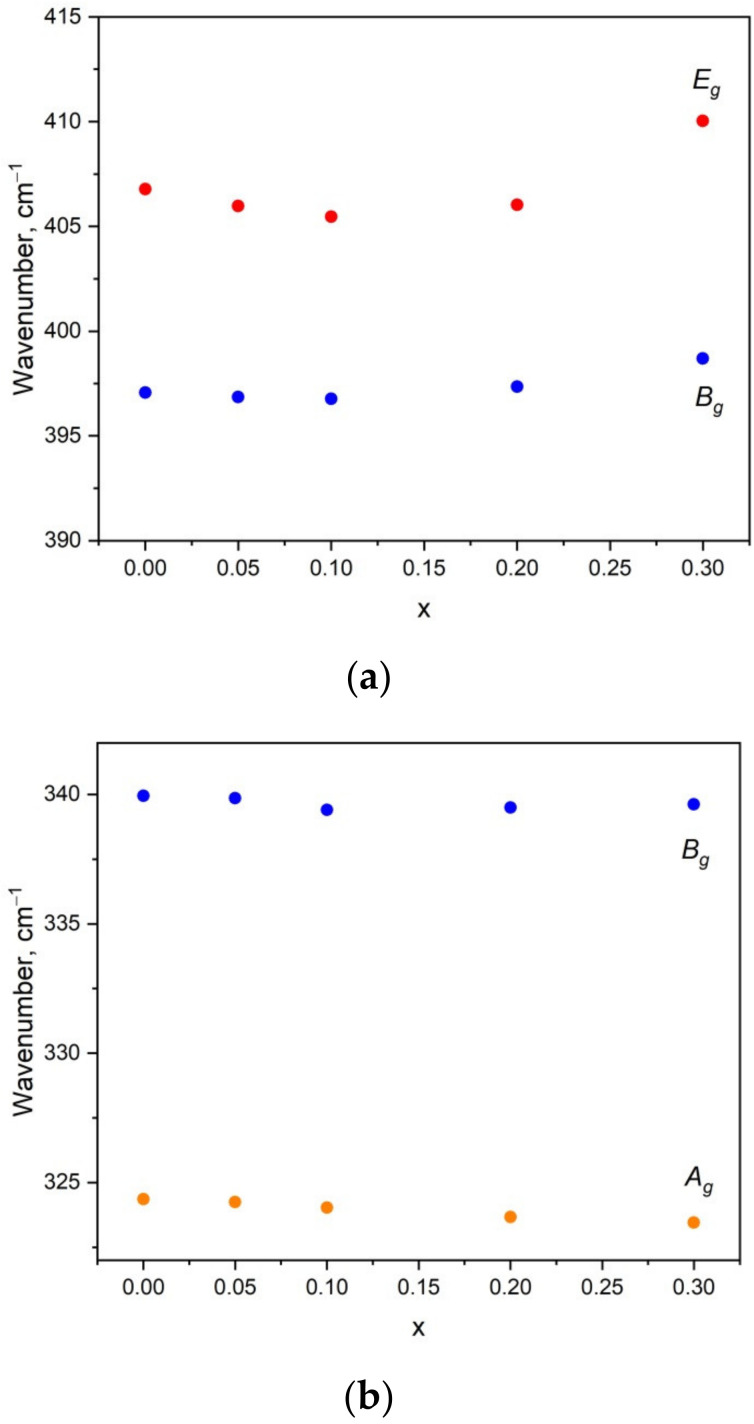

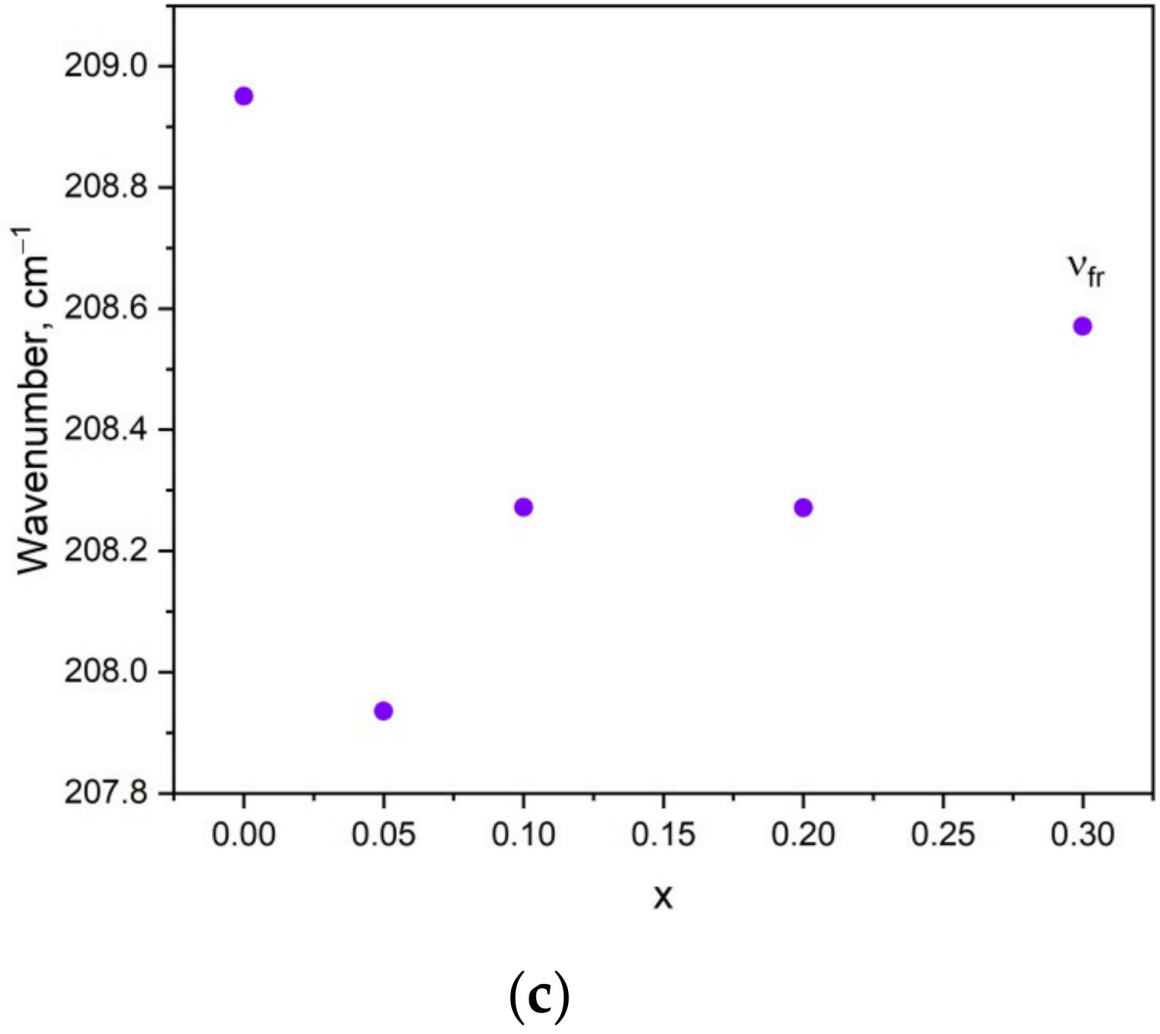
Raman shift of bands related to antisymmetric (**a**) and symmetric (**b**) bending and free rotation (**c**) of MoO_4_ tetrahedra in Li_x_Na_1-x_CaGd_0.5_(MoO_4_)_3_:Ho^3+^_0.05_/Yb^3+^_0.45_ (x = 0, 0.05, 0.1, 0.2, 0.3).

**Figure 8:**
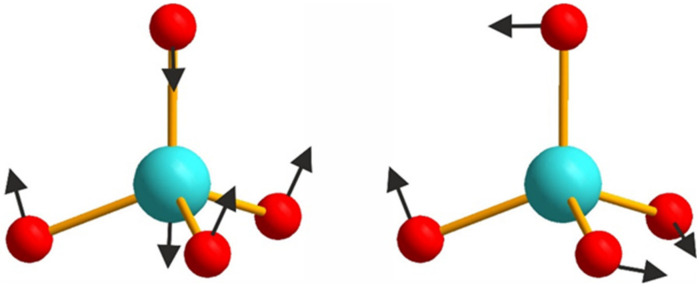
Antisymmetric (**left**) and symmetric (**right**) bending modes of MoO_4_ tetrahedra.

It is interesting to note that the Raman profiles of Li_x_Na_1-x_CaGd_0.5_(MoO_4_)_3_:Ho^3+^0.05/Yb^3+^0.45 (x= 0, 0.05, 0.1, 0.2, 0.3) in the range of free rotation and bending modes of MoO_4_ tetrahedra are sensitive to the Li content, as seen in Figure 7. The range of MoO_4_ stretching vibrations is beyond the scope of this discussion because of the overlapping of the Raman signal with the Ho^3+^ luminescence. As was mentioned earlier, the substitution of Li^+^ for Na^+^ should induce a decrease in the average ion radius IR(Li/Na/Ca/Gd/Ho/Yb) of the A position and a related unit cell volume decrease. In turn, this should lead to minor changes in the geometry of molybdenum–oxygen tetrahedra, namely, changes in bond lengths (see Table S2) and angles that should affect the Raman shift of vibration modes. The graphical representation of MoO_4_ bending vibrations is presented in Figure 8, and we can suppose that the angle variation in MoO_4_ units should affect the symmetric bending, while the bond length variation should lead to changes in the Raman shift of antisymmetric bending vibrations. Thus, we can summarize that the Li content variation in Li_x_Na_1-x_CaGd_0.5_(MoO_4_)_3_:Ho_3+_0.05/Yb^3+^0.45 leads to minor changes in the MoO_4_ bond length that are consistent with the changes in the Raman profiles in the range of antisymmetric bending vibrations. For the free MoO_4_ rotation vibration, the Raman line position variation is less than 1 cm^−1^.

**Figure 9:**
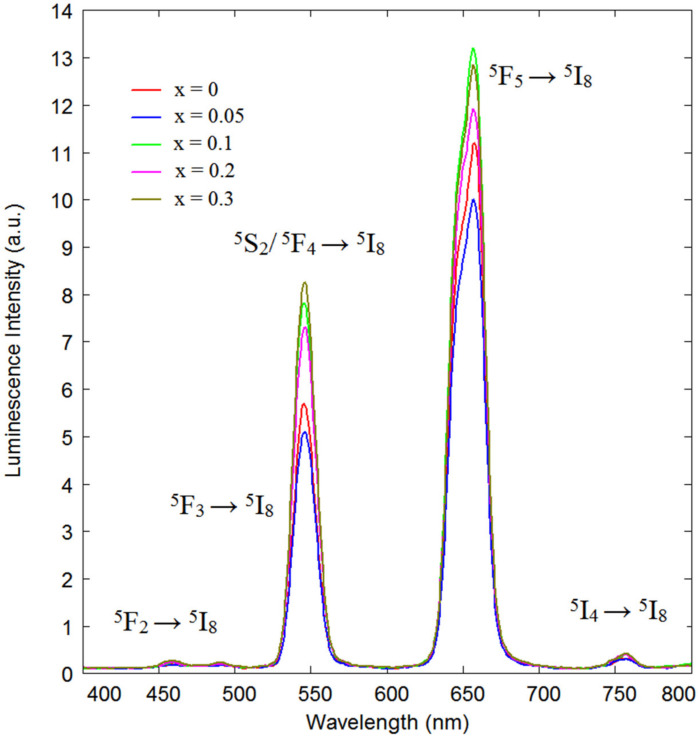
The UC photoluminescence spectra of (a) NCGM:HY, (b) LiNCGM:HY-0.05, (c) LiNCGM:HY-0.1, (d) LiNCGM:HY-0.2, and (e) LiNCGM:HY-0.3 particles excited under 980 nm at room temperature.

The UC emission spectra of Na_1-x_Li_x_CaGd(MoO_4_)_3_:Ho,Yb at x = 0, 0.05, 0.1, 0.2, and 0.3 excited at 980 nm at room temperature are shown in Figure 9. Under the excitation at 980 nm, the samples exhibited yellow emission composed of red and green emission bands of the Ho^3+^ ion, namely, the ^5^S_2_/^5^F_4_ → ^5^I_8_ band is in green and the ^5^F_5_ → ^5^I_8_ band is in red [42]. The peak intensity variation of the green and red bands, as well as the integral intensity of UC luminescence over the whole spectrum, shows its complex behavior, as seen in Figure S4, maximizing at two different Li contents of x = 0.1 and 0.3. The incorporation of Li^+^ ions into the lattice of hosts instead of ions with a larger radius is known to be efficient for controlling the luminescence of doping ions via the crystal field variation affecting these luminescing ions [76]. Li^+^ ions commonly do not destroy or alter the local symmetry of the rare earth ions in the lattice, but they can provide more appropriate UC intensities [76,77]. This is the case for the crystal structure under study. All Na, Li Ca, Gd, Ho, and Yb ions occupy the same site in the scheelite crystal structure. The introduction of Li instead of Na leads to a decrease in the tetragonal unit cell parameters a and c. However, it is a specific feature of the scheelite-type crystal structure under study that this decrease is rather tiny for x = 0.05 − 0.2, and at x = 0.3, a much stronger decrease in the unit cell size along the c axis is observed, indicating a more prominent distortion of the cell and of local structural elements within it. Specifically, all large ions mentioned above in the scheelite structure are coordinated by eight oxygen ions forming polyhedra with the local symmetry S_4_. The distances between a large cation and oxygen are not all equal, but take two unequal values, However, for the crystal under study with Li content x = 0, the difference between these values (2.484 Å) is below the accuracy of measurement by XRD. At x = 0.3, the Me–O distance values are noticeably different, being 2.44 and 2.53 Å, indicating a distortion of the large cation polyhedron. An additional factor that may be at work in the case of the scheelite structure is the noticeable electric field due to the stochastic cation distribution over equivalent sites. This factor may lead to variability of the cation distribution characteristics upon the introduction of Li ions. The existence of several possible mechanisms working as the Li content is varied produces, in our opinion, the complex dependence of the UC luminescence in Figure 9. The similarity of this dependency for both green and red bands means, as we suggest, that the introduction of Li influences the two-stage UC excitation channel, while the de-excitation channel probability variations play a less important role, resulting in minor changes in the relative intensities of red and green bands.

It is interesting to compare the behavior of LiNCGM:HY studied in this paper and that of LiNCLM:HY [67] at a different Li content. In the case of LiNCLM:HY, the green upconversion band experiences two maxima, while the red upconversion band experiences a single maximum at the Li content x = 0.2. The explanation for the different behavior of the two hosts arises from the different ionic radii of La and Gd. Ho and Yb ions experience, in general, a stronger crystal field when they occupy the Gd position than when they occupy the La position in the crystal structure of the host. As a result, the LiNCGM:HY behavior is more complex in the case of varied Li content.

**Figure 10:**
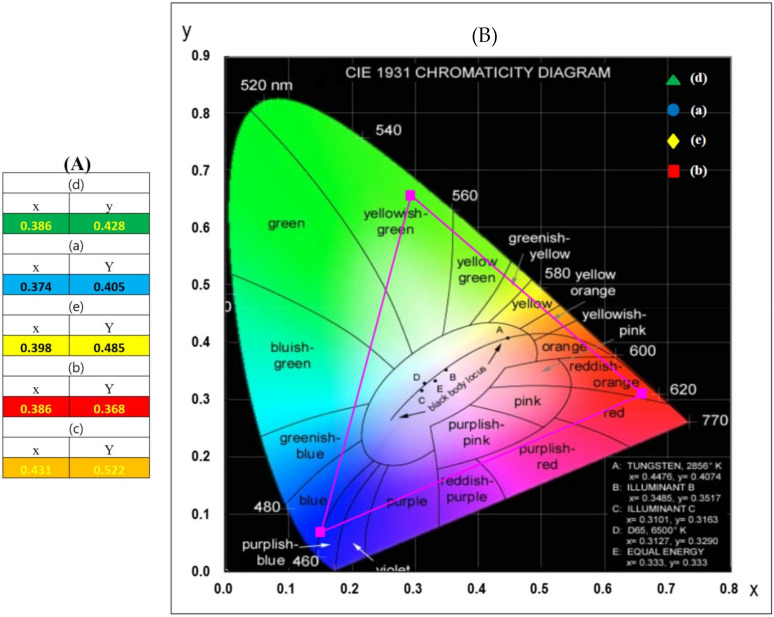
(**A**) CIE chromaticity diagram for the LiNCGM:HY phosphors and (**B**) calculated chromaticity coordinate (x, y) values. The emission points for the samples are shown with the legend.

The CIE diagram and individual chromaticity points (ICP) for (*x*, *y*) of LiNCGM:HY phosphors are shown in Figure 10. The ICP of the CIE for samples (a), (b), (c), and (d) are exhibited by the legend in Figure 10A. The calculated values for chromaticity coordinates are *x* = 0.374 and *y* = 0.409 for (a), *x* = 0.486 and *y* = 0.368 for (b)_,_ *x* = 0.431 and *y* = 0.522 for (c), and *x* = 0.386, and *y* = 0.428 for (d), corresponding to the equal-energy point in the standard CIE diagram. As can be seen, LiNCGM:HY phosphors provide emission in the yellowish region.

## 4. Conclusions

New MAS-derived triple molybdate LiNCGM:HY phosphors under the variation of Li_x_Na_1-x_ (x = 0, 0.05, 0.1, 0.2, 0.3, 0.4) were successfully manufactured. The resultant particles after annealing at 800 °C for 16 h provided well-crystallized ST tetragonal phases with particles of size 3–10 μm. The crystal structures of LiNCGM:HY phosphors at room temperature were determined in space group *I*4_1_/*a* by Rietveld analysis. NCGM:HY has a scheelite-type structure with cell parameters *a* = 5.24782 (11) and *c* = 11.5107 (3) Å, *V* = 317.002 (17) Å^3^, *Z* = 4. In doped samples, the sites are occupied by a mixture of (Li,Na,Gd,Ho,Yb) ions, and this provides a linear cell volume decrease with increasing doping level. Under the excitation derived from 980 nm, the final particles led to the formation of yellow emissions based on the ^5^S_2_/ ^5^F_4_ → ^5^I_8_ green emission and the ^5^F_5_ → ^5^I_8_ red emission. The incorporated Li^+^ ions gave rise to local symmetry distortion around the cations in the substituted crystal structure by the Ho^3+^ and Yb^3+^ ions, and further affected the UC transition probabilities in the quadruple molybdate of LiNCGM:HY. The Raman spectra of LiNCGM doped with Ho^3+^ and Yb^3+^ ions were totally covered by the luminescence signal of Ho^3+^ ions, and increasing the Li content resulted in a difference in the multiplet Ho^3+^ intensity. Variation of Raman line positions was observed in the range of MoO_4_ bending vibrations depending on the Li content.

## Figures and Tables

**Table 1:** Abbreviations used as sample notation for Li_x_Na_1-x_CaGd(MoO_4_)_3_:Ho^3+^_0.05_/Yb^3+^_0.45_.

Scheme 0.	Chemical Composition
NCGM:HY	NaCaGd_0.5_Ho_0.05_Yb_0.45_(MoO_4_)_3_
LiNCGM:HY-0.05	Li_0.05_Na_0.95_CaGd_0.5_Ho_0.05_Yb_0.45_(MoO_4_)_3_
LiNCGM:HY-0.1	Li_0.1_Na_0.9_CaGd_0.5_Ho_0.05_Yb_0.45_(MoO_4_)_3_
LiNCGM:HY-0.2	Li_0.2_Na_0.8_CaGd_0.5_Ho_0.05_Yb_0.45_(MoO_4_)_3_
LiNCGM:HY-0.3	Li_0.3_Na_0.7_CaGd_0.5_Ho_0.05_Yb_0.45_(MoO_4_)_3_

**Table 2:** Main parameters of processing and refinement of the Li_x_Na_1-x_CaGd_0.5_Ho_0.05_Yb_0.45_(MoO_4_)_3_ samples.

x.	0.	0.05.	0.1.	0.2.	0.3.
Sp.Gr.	*I*4_1_/*a*	*I*4_1_/*a*	*I*4_1_/*a*	*I*4_1_/*a*	*I*4_1_/*a*
*a*, Å	5.2077 (2)	5.2074 (2)	5.20679 (11)	5.20342 (11)	5.19940 (8)
*c*, Å	11.3657 (5)	11.3615 (6)	11.3597 (3)	11.3508 (3)	11.3043 (2)
*V*, Å^3^	308.24 (3)	308.09 (3)	307.968 (15)	307.329 (15)	306.572 (12)
*Z*	4	4	4	4	4
*2θ* interval, º	10–90	10–90	10–90	10–90	10–90
No. of reflections	63	63	63	63	63
No. of refined parameters	7	7	7	7	7
*R_wp_*, %	19.79	18.39	15.48	15.82	15.44
*R_p_*, %	14.58	13.00	10.50	10.98	10.59
*R_ex__p_*, %	14.03	14.37	13.93	13.38	13.63
*χ* *^2^*	1.41	1.28	1.11	1.18	1.13
*R_B_*, %	7.59	3.66	2.19	2.82	1.41

## Supplementary Materials

The following are available online, Figure S1: Difference Rietveld plots of LiNCGM:HY-0.05, Figure S2: Difference Rietveld plots of LiNCGM:HY-0.1, Figure S3: Difference Rietveld plots of LiNCGM:HY-0.2, Figure S4: Variations of integral intensity and intensity of specific UC bands upon Li content, Table S1: Fractional atomic coordinates and isotropic displacement parameters (Å2) of LiNCGM:HY samples, Table S2: Main bond lengths (Å) of LiNCGM:HY samples, Table S3: Main parameters of processing and refinement of the NaCaGd(MoO_4_)_3_:xEr,yYb samples [64], Table S4: Main parameters of processing and refinement of the NaCaGd(MoO_4_)_3_:xHo,yYb samples [65], Table S5: Main parameters of processing and refinement of the Li_x_Na_1-x_CaLa(MoO_4_)_3_:0.05Ho,0.45Yb samples [66], related cif files.
