# Supplementary material for: Structural and Spectroscopic Effects of Li+ Substitution for Na+ in LixNa1-xCaGd0.5Ho0.05Yb0.45(MoO4)3 Scheelite-Type Upconversion Phosphors
Source: Molecules. 2021 Dec 3;26(23):7357. doi: 10.3390/molecules26237357 (PMC8659099; doi:10.3390/molecules26237357)
Supplement: Supplementary file 1 [file molecules-26-07357-s001.zip › Support/LiNaCaGd(MoO4)3 Ho3,Yb3_Support_02_12_2021.pdf]

Supporting Information

Structural and spectroscopic effects of Li^+ substitution for Na^+ in $Li_xNa_{1-x}CaGd_{0.5}Ho_{0.05}Yb_{0.45}(MoO_4)_3$ scheelite-type upconversion phosphors

Chang Sung Lim [1,*,†], Aleksandr Aleksandrovsky [2,3], Maxim Molokeev [4,5,6], Aleksandr Oreshonkov [7,8] and Victor Atuchin [9,10,11,*,† †]

[1]Department of Aerospace Advanced Materials and Chemical Engineering, Hanseo University, Seosan 31962, Korea

[2]Laboratory of Coherent Optics, Kirensky Institute of Physics Federal Research Center KSC SB RAS, Krasnoyarsk 660036, Russia

[3]Institute of Nanotechnology, Spectroscopy and Quantum Chemistry, Siberian Federal University, Krasnoyarsk 660041, Russia

[4]Laboratory of Crystal Physics, Kirensky Institute of Physics, Federal Research Center KSC SB RAS, Krasnoyarsk 660036, Russia

[5]Institute of Engineering Physics and Radioelectronics, Siberian Federal University, Krasnoyarsk 660041, Russiakrasn.ru

[6]Department of Physics, Far Eastern State Transport University, Khabarovsk 680021, Russia

[7]Laboratory of Molecular Spectroscopy, Kirensky Institute of Physics, Federal Research Center KSC SB RAS, Krasnoyarsk 660036, Russia

[8]School of Engineering and Construction, Siberian Federal University, Krasnoyarsk 660041, Russia

[9]Laboratory of Optical Materials and Structures, Institute of Semiconductor Physics, SB RAS, Novosibirsk 630090, Russia

[10]Research and Development Department, Kemerovo State University, Kemerovo 650000, Russia

[11]Department of Industrial Machinery Design, Novosibirsk State Technical University, Novosibirsk 630073, Russia

*Correspondence: cslim@hanseo.ac.kr (C.S.L.); atuchin@isp.nsc.ru (V.V.A.)

†Hanseo University, Seosan 31962, Korea

††Institute of Semiconductor Physics, SB RAS, Novosibirsk 630090, Russia

Table S1. Fractional atomic coordinates and isotropic displacement parameters (Å^2) of LiNCGM:HY samples

Atom	*x*	*y*	*z*	B_{iso}	Occ.
		$Na_{1/3}Ca_{1/3}Gd_{1/6}MoO_4$:0.05Ho,0.45Yb			
Na	0	0.25	0.625	1.89 (19)	1/3
Ca	0	0.25	0.625	1.89 (19)	1/3
Gd	0	0.25	0.625	1.89 (19)	1/6
Ho	0	0.25	0.625	1.89 (19)	1/6
Yb	0	0.25	0.625	1.89 (19)	0.15
Mo	0	0.25	0.125	1.64 (19)	1
O	0.222 (2)	0.1182 (15)	0.0338 (7)	2.4 (3)	1
		$Na_{0.95/3}Li_{0.05/3}Ca_{1/3}Gd_{1/6}MoO_4$:0.05Ho,0.45Yb			
Na	0	0.25	0.625	0.49 (19)	0.95/3
Li	0	0.25	0.625	0.49 (19)	0.05/3
Ca	0	0.25	0.625	0.49 (19)	1/3
Gd	0	0.25	0.625	0.49 (19)	1/6
Ho	0	0.25	0.625	0.49 (19)	1/60
Yb	0	0.25	0.625	0.49 (19)	0.15
Mo	0	0.25	0.125	0.64 (18)	1
O	0.242 (2)	0.1003 (14)	0.0389 (6)	0.6 (3)	1
		$Na_{0.9/3}Li_{0.1/3}Ca_{1/3}Gd_{1/6}MoO_4$:0.05Ho,0.45Yb			
Na	0	0.25	0.625	0.32 (17)	0.9/3
Li	0	0.25	0.625	0.32 (17)	0.1/3
Ca	0	0.25	0.625	0.32 (17)	1/3
Gd	0	0.25	0.625	0.32 (17)	1/6
Ho	0	0.25	0.625	0.32 (17)	1/60
Yb	0	0.25	0.625	0.32 (17)	0.15
Mo	0	0.25	0.125	0.50 (17)	1
O	0.2404 (18)	0.1030 (11)	0.0401 (5)	0.5 (3)	1
		$Na_{0.8/3}Li_{0.2/3}Ca_{1/3}Gd_{1/6}MoO_4$:0.05Ho,0.45Yb			
Na	0	0.25	0.625	0.72 (15)	0.8/3
Li	0	0.25	0.625	0.72 (15)	0.2/3
Ca	0	0.25	0.625	0.72 (15)	1/3
Gd	0	0.25	0.625	0.72 (15)	1/6
Ho	0	0.25	0.625	0.72 (15)	1/60
Yb	0	0.25	0.625	0.72 (15)	0.15
Mo	0	0.25	0.125	0.69 (14)	1

O	0.2472 (19)	0.1028 (12)	0.0392 (5)	1.1 (2)	1
$Na_{0.7/3}Li_{0.3/3}Ca_{1/3}Gd_{1/6}MoO_4$:0.05Ho,0.45Yb					
Na	0	0.25	0.625	1.03 (14)	0.7/3
Li	0	0.25	0.625	1.03 (14)	0.3/3
Ca	0	0.25	0.625	1.03 (14)	1/3
Gd	0	0.25	0.625	1.03 (14)	1/6
Ho	0	0.25	0.625	1.03 (14)	1/60
Yb	0	0.25	0.625	1.03 (14)	0.15
Mo	0	0.25	0.125	1.08 (14)	1
O	0.2406 (18)	0.1065 (11)	0.0418 (5)	1.3 (2)	1

Table S2. Main bond lengths (Å) of LiNCGM:HY samples

$Na_{1/3}Ca_{1/3}Gd_{1/6}MoO_4$:0.05Ho,0.45Yb			
(Na/Ca/Gd/Ho/Yb)—O^{i}	2.616 (10)	Mo—O	1.699 (10)
(Na/Ca/Gd/Ho/Yb)—O^{ii}	2.413 (9)		
$Na_{0.95/3}Li_{0.05/3}Ca_{1/3}Gd_{1/6}MoO_4$:0.05Ho,0.45Yb			
(Na/Li/Ca/Gd/Ho/Yb)—O^{i}	2.467 (10)	Mo—O	1.776 (9)
(Na/Li/Ca/Gd/Ho/Yb)—O^{ii}	2.425 (8)		
$Na_{0.9/3}Li_{0.1/3}Ca_{1/3}Gd_{1/6}MoO_4$:0.05Ho,0.45Yb			
(Na/Li/Ca/Gd/Ho/Yb)—O^{i}	2.477 (8)	Mo—O	1.756 (8)
(Na/Li/Ca/Gd/Ho/Yb)—O^{ii}	2.436 (7)		
$Na_{0.8/3}Li_{0.2/3}Ca_{1/3}Gd_{1/6}MoO_4$:0.05Ho,0.45Yb			
(Na/Li/Ca/Gd/Ho/Yb)—O^{i}	2.459 (8)	Mo—O	1.786 (8)
(Na/Li/Ca/Gd/Ho/Yb)—O^{ii}	2.406 (7)		
$Na_{0.7/3}Li_{0.3/3}Ca_{1/3}Gd_{1/6}MoO_4$:0.05Ho,0.45Yb			
(Na/Li/Ca/Gd/Ho/Yb)—O^{i}	2.479 (8)	Mo—O	1.735 (8)
(Na/Li/Ca/Gd/Ho/Yb)—O^{ii}	2.440 (7)		

Symmetry codes: (i) -*x*+1/2, -*y*, *z*+1/2; (ii) -*x*+1/2, -*y*+1/2, -*z*+1/2.

Table S3. Main parameters of processing and refinement of the $NaCaGd(MoO_4)_3$:*x*Er,*y*Yb samples [64]

x, y	0, 0	0.2, 0	0.1, 0.2	0.05, 0.45
Sp.Gr.	$I4_1/a$	$I4_1/a$	$I4_1/a$	$I4_1/a$
a, Å	5.22881 (14)	5.22313 (11)	5.21690 (13)	5.20680 (13)
c, Å	11.4228 (3)	11.4095 (3)	11.3911 (3)	11.3635 (4)
V, Å^3	312.304 (18)	311.263 (15)	310.02 (2)	308.08 (2)
Z	4	4	4	4
2θ-interval, ^{o}	10-90	10-90	10-90	10-90
No. of reflections	67	67	67	67
No. of refined parameters	7	7	7	7
R_{wp}, %	17.88	18.175	16.89	16.05
R_p, %	11.67	12.62	11.26	10.76
R_{exp}, %	15.97	15.84	15.15	14.00
χ^2	1.12	1.15	1.11	1.15
R_B, %	3.15	2.89	2.37	2.43

Table S4. Main parameters of processing and refinement of the $NaCaGd(MoO_4)_3$:*x*Ho,*y*Yb samples [65]

x, y	0, 0	0.05, 0.35	0.05, 0.40	0.05, 0.45	0.05, 0.50
Sp.Gr.	$I4_1/a$	$I4_1/a$	$I4_1/a$	$I4_1/a$	$I4_1/a$
a, Å	5.2287 (1)	5.2125 (2)	5.20938 (19)	5.20933 (11)	5.20550 (11)
c, Å	11.4226 (2)	11.3799 (5)	11.3720 (4)	11.3666 (3)	11.3613 (3)
V, Å^3	312.291 (13)	309.20 (3)	308.61 (3)	308.457 (16)	307.859 (16))
Z	4	4	4	4	4
2θ-interval, ^{o}	10-90	10-90	10-90	10-90	10-90
No. of reflections	67	67	67	67	67
No. of refined parameters	7	7	7	7	7
R_{wp}, %	18.49	18.14	15.91	15.07	14.88
R_p, %	12.62	12.91	10.84	10.63	10.16
R_{exp}, %	15.96	14.19	13.99	13.38	13.13
χ^2	1.16	1.28	1.14	1.13	1.13
R_B, %	3.20	2.59	2.18	1.61	2.07

Table S5. Main parameters of processing and refinement of the $Li_xNa_{1-x}CaLa(MoO_4)_3$:0.05Ho,0.45Yb samples [66]

x	0	0.05	0.1	0.2	0.3
Sp.Gr.	$I4_1/a$	$I4_1/a$	$I4_1/a$	$I4_1/a$	$I4_1/a$
a, Å	5.2451 (3)	5.24541 (14)	5.24314 (17)	5.2447 (2)	5.2404 (3)
c, Å	11.4741 (7)	11.4743 (4)	11.4656 (5)	11.4729 (5)	11.4682 (8)
V, Å^3	315.67 (4)	315.71 (2)	315.20 (2)	315.58 (3)	314.93 (4)
Z	4	4	4	4	4
2θ-interval, °	10-90	10-90	10-90	10-90	10-90
No. of reflections	67	67	67	67	67
No. of refined parameters	7	7	7	7	7
R_{wp}, %	15.87	17.45	17.43	19.64	16.96
R_p, %	10.88	12.52	12.13	13.44	11.89
R_{exp}, %	13.43	14.47	15.17	14.73	15.06
χ^2	1.18	1.21	1.15	1.33	1.13
R_B, %	2.50	4.40	2.91	4.13	3.03

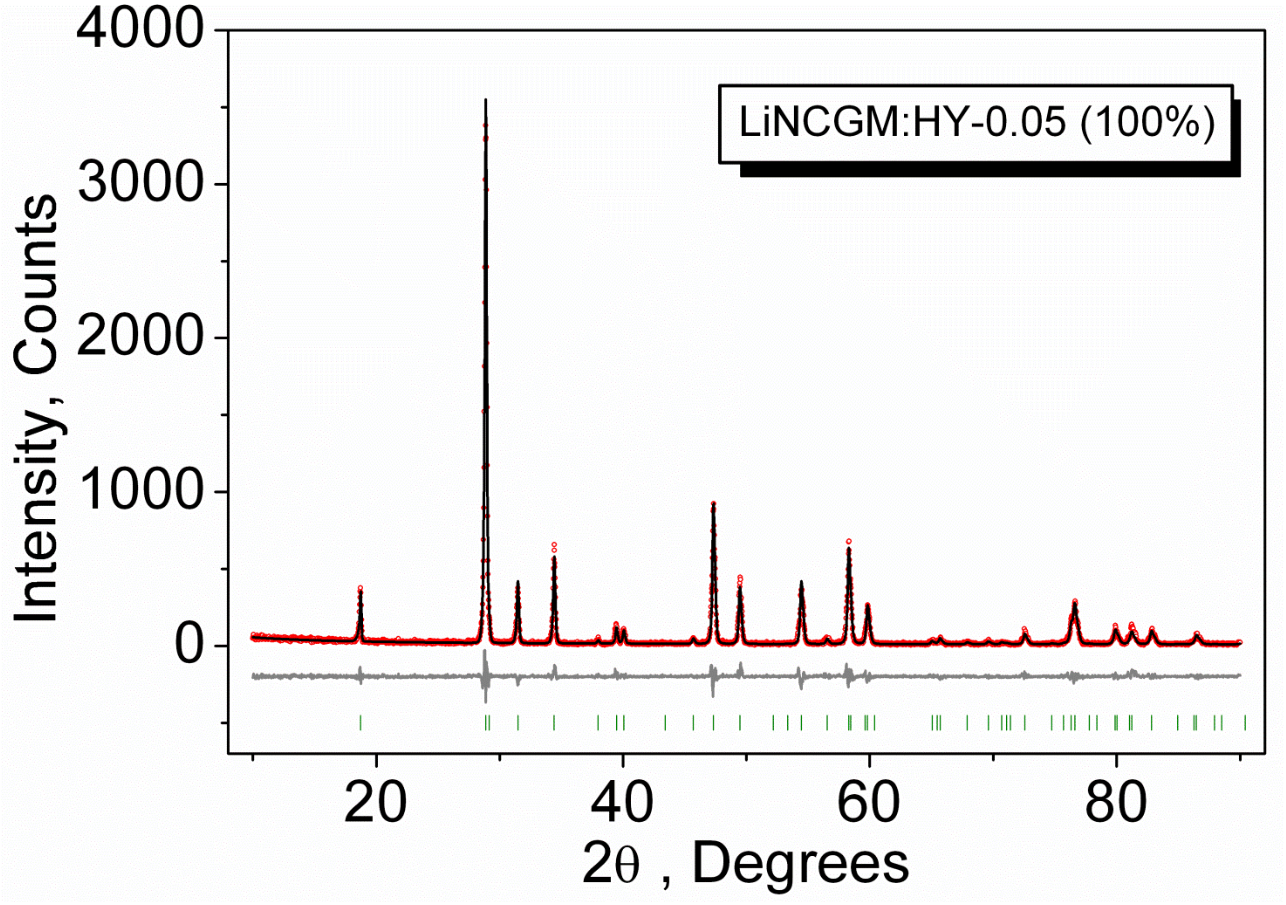

Figure S1. Difference Rietveld plots of LiNCGM:HY-0.05.

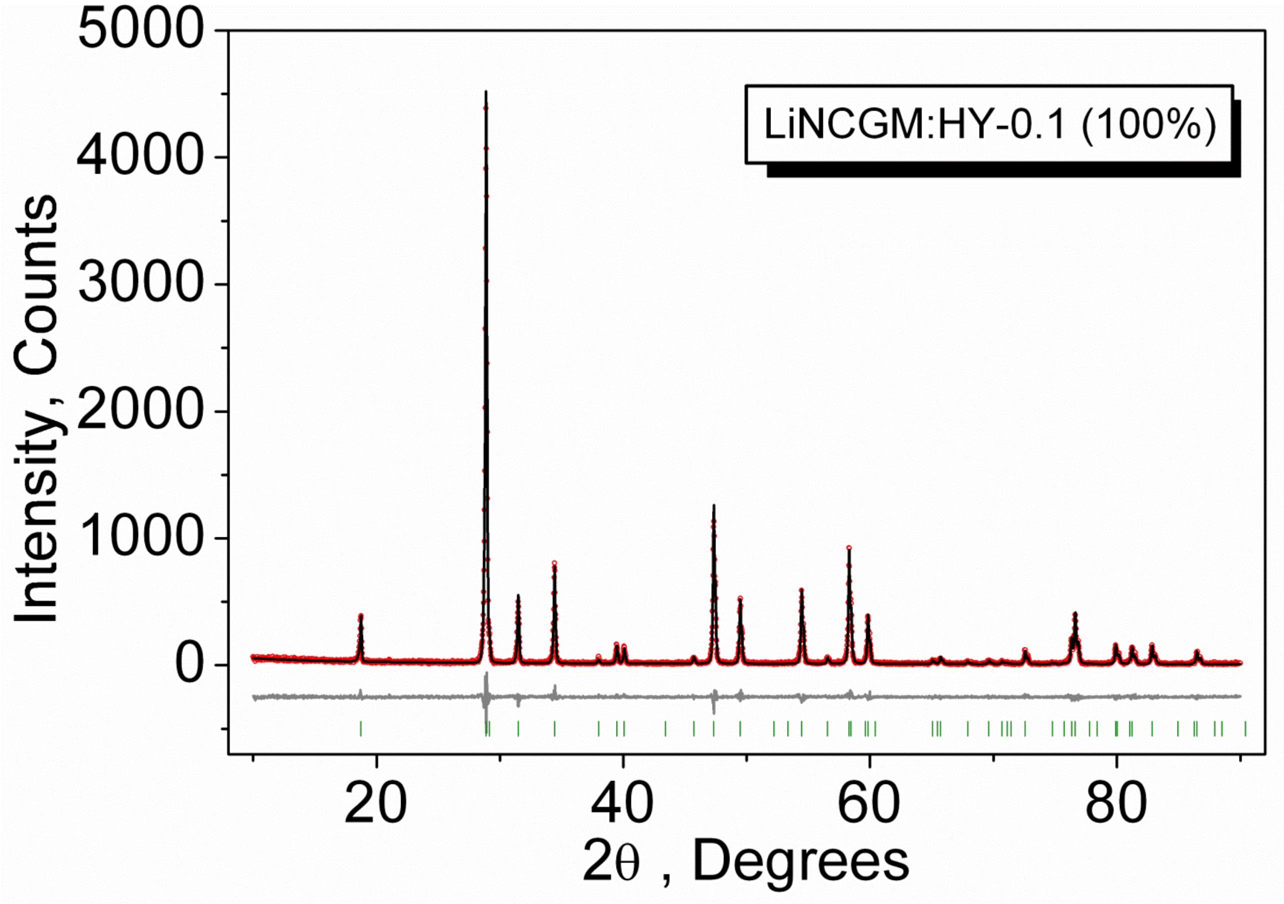

Figure S2. Difference Rietveld plots of LiNCGM:HY-0.1.

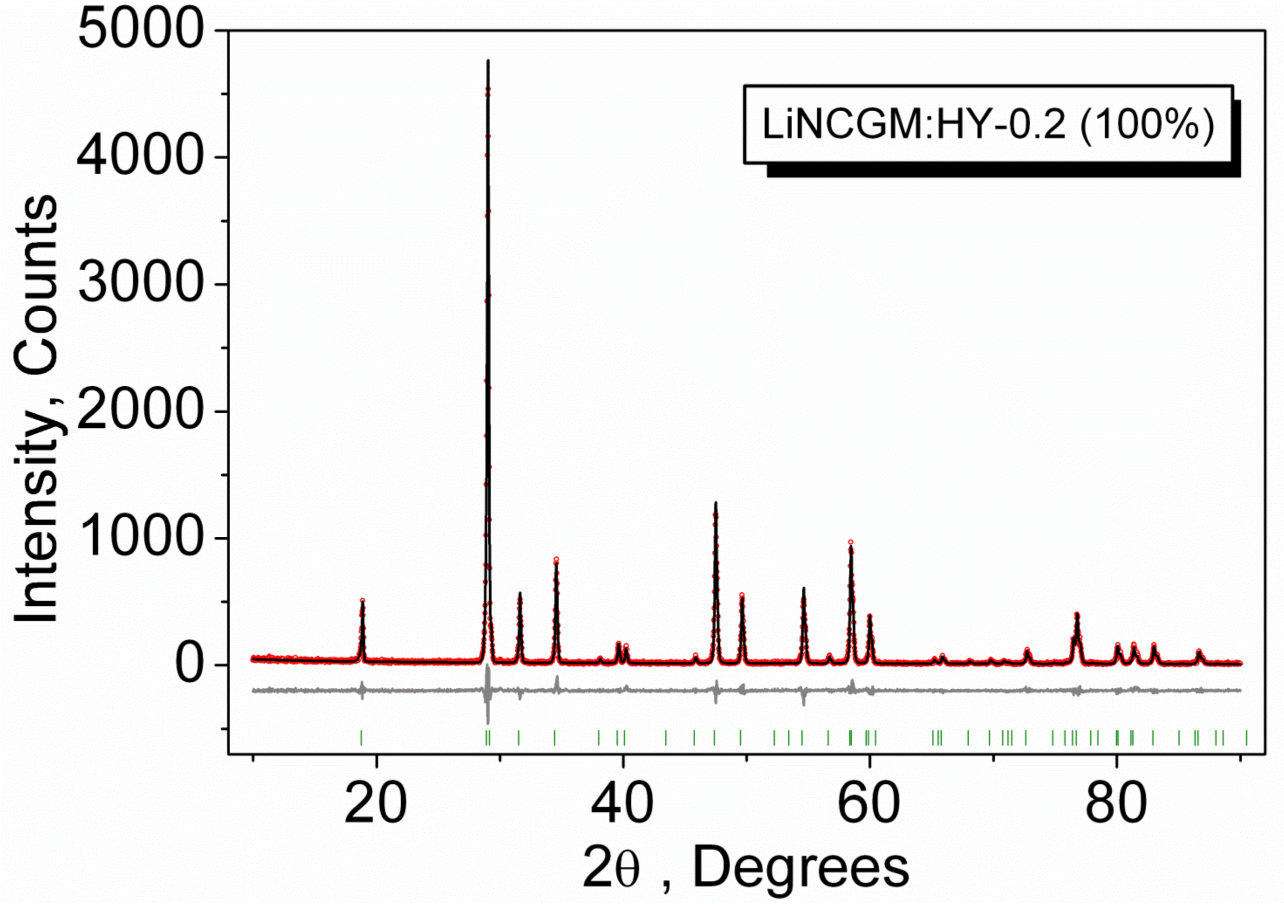

Figure S3. Difference Rietveld plots of LiNCGM:HY-0.2.

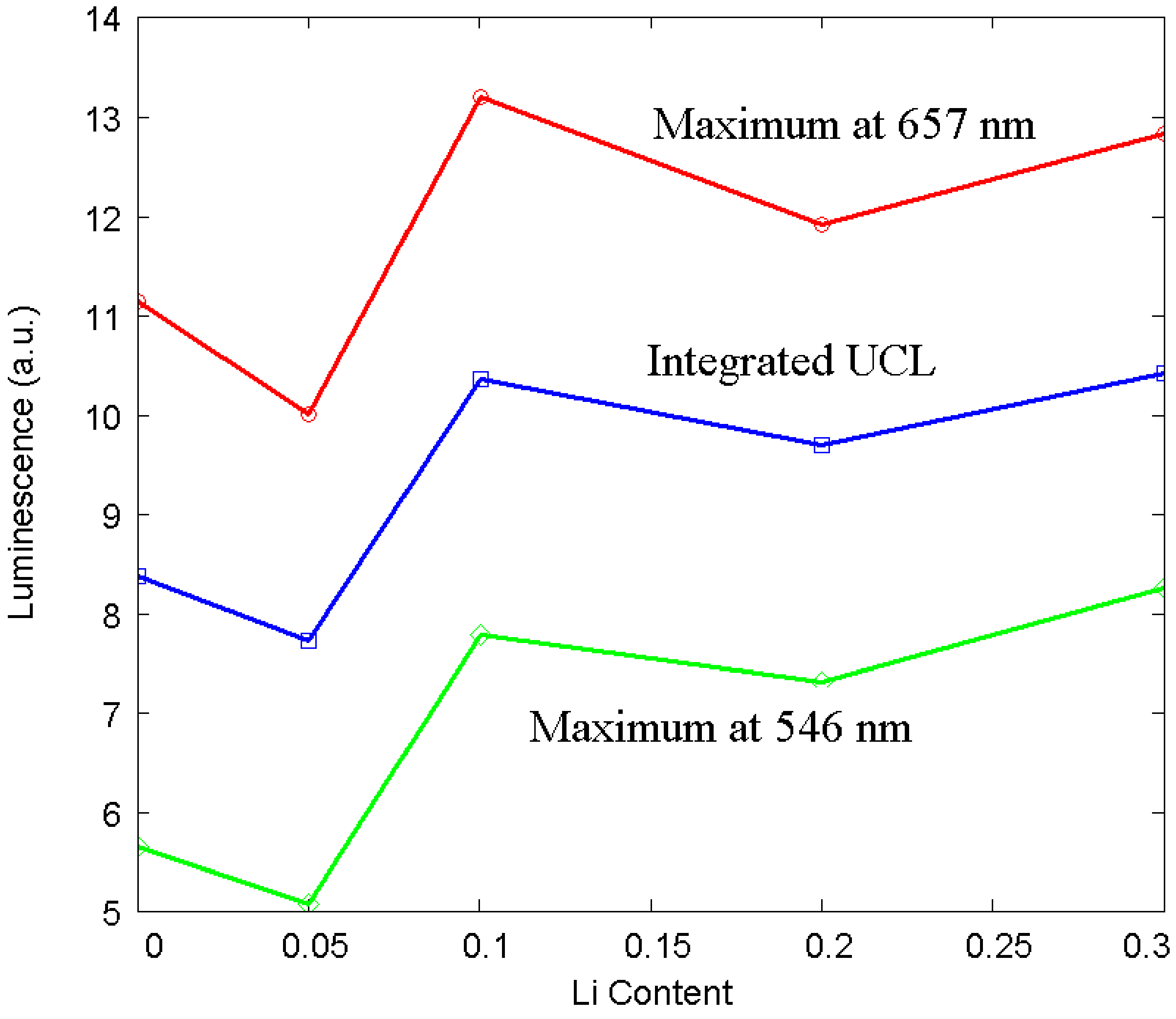

Figure S4. Variations of integral intensity and intensity of specific UC bands upon Li content.
